# Untreated Anemia in Nontransfusion-dependent β-thalassemia: Time to Sound the Alarm

**DOI:** 10.1097/HS9.0000000000000806

**Published:** 2022-11-15

**Authors:** Khaled M. Musallam, Ali T. Taher, Maria Domenica Cappellini, Olivier Hermine, Kevin H. M. Kuo, Sujit Sheth, Vip Viprakasit, John B. Porter

**Affiliations:** 1 Thalassemia Center, Burjeel Medical City, Abu Dhabi, United Arab Emirates; 2 Department of Internal Medicine, American University of Beirut Medical Center, Beirut, Lebanon; 3 Department of Clinical Sciences and Community, University of Milan, Ca’ Granda Foundation IRCCS Maggiore Policlinico Hospital, Milan, Italy; 4 Department of Hematology, Necker Hospital, Paris, France; 5 Division of Hematology, University of Toronto, ON, Canada; 6 Division of Hematology and Oncology, Department of Pediatrics, Weill Cornell Medicine, New York, NY, USA; 7 Department of Pediatrics & Thalassemia Center, Faculty of Medicine, Siriraj Hospital, Mahidol University, Bangkok, Thailand; 8 Department of Haematology, University College London, UK

Despite advances in management and the consequent improvement in survival, a diagnosis of β-thalassemia continues to confer considerable burden on patients, their families, and the healthcare system.^1^ This is primarily driven by the complexity of management, including regular transfusion and iron chelation, the chronicity of the disease, need for comprehensive monitoring, and multidisciplinary care; complicated by poor response/tolerance, adherence, or access to treatment.^2,3^ Although curative therapies such as bone marrow transplantation and gene manipulation techniques are available, wide spread use of these is impeded by the need for specialized centers and expertise, strict subject or donor selection, and high cost. The future outlook for disease control and prevention of morbidity and mortality when curative therapy is not available or practical will fall to optimizing the use of conventional and supportive therapies, novel disease modifying agents, and/or their combination.^4^

The underlying basis of disease in patients with clinically morbid forms of β-thalassemia is ineffective erythropoiesis which leads to chronic anemia of varying severity, extramedullary hematopoiesis, iron overload, hypercoagulability, and their respective complications (Figure 1). The clinical severity of the disease depends on the β-globin gene mutations inherited in homozygous or compound heterozygous forms, as well as secondary genetic modifiers that can ameliorate (eg, α-globin gene deletions or mutations) or worsen (eg, α-globin gene duplications) the α/β-globin chain imbalance, leading to ineffective erythropoiesis characterized by the expansion of early erythroid progenitors and premature cell death of late erythroid precursors and red cells through various cellular pathways.^1,4^ Although no formal diagnostic criteria have ever been validated or uniformly used, patients have historically been assigned the phenotypes β-thalassemia intermedia or major based on severity of anemia and need for regular transfusion therapy, among other factors. In more recent years, the descriptors nontransfusion-dependent β-thalassemia (NTDT) and transfusion-dependent β-thalassemia (TDT) have been more commonly used in view of the considerable role of transfusion therapy (or lack thereof) in modifying the underlying pathophysiology and complications, especially as relates to iron overload and the need for iron chelation therapy. Although clinical care and research in TDT have been quite well established for the past few decades, comprehensive longitudinal data on disease burden and unmet needs in patients with NTDT have more recently been systematically analyzed.^5^

**Figure 1.**
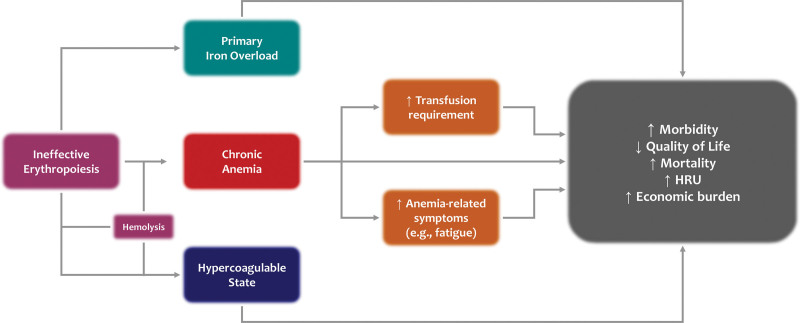
**The central role of anemia in determining outcomes in nontransfusion-dependent β-thalassemia.** HCRU = healthcare resource utilization.

Compared to patients with TDT, patients with NTDT usually present to medical attention beyond the age of 2 years, with mild-to-moderate anemia usually in the range of 7–10 g/dL of hemoglobin. It is important to note that transfusion-independence in these patients is usually a joint decision between the patient/family and the physician, as there has been an overwhelming belief that patients can thrive and survive with such hemoglobin levels, especially in early childhood where some data point to good tolerance to chronic anemia.^5,6^ In such individuals, transfusions have been commonly reserved for situations of acute stress or an anticipated drop in the hemoglobin level such as during infection, pregnancy, or surgery. Some physicians may prefer to introduce more regular transfusions for NTDT patients, especially to promote growth during childhood or to manage specific morbidities in older patients.^7^ When such practices were reviewed in retrospective observational studies, it was well-established that patients with NTDT who received regular transfusion therapy by reducing ineffective erythropoiesis and improving tissue oxygenation had improved growth, lower prevalence of several morbidities (thromboembolic disease, pulmonary hypertension, leg ulcers, extramedullary hematopoiesis), and improved survival compared to those who did not.^8–10^ This was already an indication that anemia in this patient population should not be left untreated and several subsequent studies further support this conclusion.

In one cross-sectional study of untreated NTDT patients, a receiver operator characteristic curve analysis identified a hemoglobin level of >10 g/dL as the best predictor for the absence of morbidity (liver disease, extramedullary hematopoiesis, endocrine and bone disease, leg ulcers, thrombosis, pulmonary hypertension). In a retrospective cohort study following untreated NTDT patients for 10 years, a hemoglobin level <10 g/dL was associated with a significantly worse morbidity-free survival than those with hemoglobin levels ≥10 g/dL (~4-fold increase in hazard ratio on multivariate analysis).^11^ In another study analyzing a global cohort of patients, overall survival was significantly worse in patients with a hemoglobin level ≤10 g/dL than those with >10 g/dL (~8-fold increase in hazard ratio on multivariate analysis).^12^ Low hemoglobin levels have also been linked to cerebrovascular disease in NTDT patients.^13^ Interestingly, increases by 1 g/dL of hemoglobin (interpatient variation) were found to significantly ameliorate the risk of adverse morbidity outcomes.^14^ Collectively, these findings are not surprising considering the impact of chronic anemia and hypoxia on tissue health in various organs. More importantly, anemia is a direct and the most easily attainable marker of ineffective erythropoiesis, which we now know can lead to increased iron absorption and primary iron overload and subsequent hepatic, endocrine, renal, and vascular disease in NTDT.^15^ Erythroid cells released through ineffective erythropoiesis have also been shown to possess thrombogenic properties that increase hypercoagulability, which among other factors, lead to high rates of thromboembolic and vascular events in NTDT patients, especially those who are splenectomized.^16^ In addition to increased risks of long-term morbidity and mortality, chronic anemia can also impact patients’ well-being in the short term with symptoms of fatigue and decreased exercise tolerance, leading to poor quality of life and mental health^17–19^—findings that are also noted in individuals without thalassemia.^20^

The key practical questions that follow are which patients should be treated for anemia, starting when, and with what therapy? Ideally, all patients with hemoglobin levels 10 g/dL or less should be considered, especially those who are symptomatic or at high-risk/with history of morbidity. Earlier management starting in childhood could promote growth and prevent the development of clinical complications in adulthood. Transfusions are the only available treatment option, but the major concern is the substitution of untreated thalassemia complications with a new set of complications, related to transfusional iron overload.^4^ Splenectomy has been used in the past to improve hemoglobin levels, but recent evidence suggests that other than infections, splenectomy is linked to a high risk of thromboembolic events.^8^ Experiences with several old agents such as hydroxyurea have been reported in case series and small trials, but results were not always encouraging and limited to subsets of patients with specific molecular profiles from certain geographic regions.^21^

Today, the hope for a treatment option targeting anemia in NTDT is tied to various novel agents currently in clinical development. All programs are primarily investigating efficacy and safety in adults with hemoglobin levels ≤10–11 g/dL. These include the erythroid maturation agent luspatercept, the pyruvate kinase activators mitapivat and etavopivat, stimulators of hepcidin production (the antisense oligonucleotide TMPRSS6-LRx and small interfering ribonucleic acid SLN124), and the ferroportin inhibitor VIT-2763^22^; all that have shown amelioration of ineffective erythropoiesis or an increase in erythroid cell lifespan in animal models and are at different stages of clinical development, with luspatercept and mitapivat leading the way with final data available from phase 2 trials.^23,24^

Unfortunately, the first to approach regulatory approval in the United States for the treatment of anemia in NTDT had its application withdrawn in June 2022 for the lack of agreement on benefit/risk.^25^ The submission was based on data from the BEYOND trial, a phase 2 randomized placebo-controlled trial evaluating the efficacy and safety of luspatercept in adult patients with NTDT and a hemoglobin of ≤10 g/dL.^24^ The trial met its primary endpoint of an increase from baseline of ≥1 g/dL in mean hemoglobin level over a continuous 12-week interval during weeks 13–24, in the absence of transfusions (response of 77% in luspatercept versus 0% in placebo). The key secondary endpoint of improvement in a patient-reported outcome measure of tiredness and weakness (NTDT-PRO) was in favor of luspatercept, although without statistical significance. Improvement in NTDT-PRO became more pronounced over time and when considering symptomatic patients at baseline. Safety was largely similar with those observed in previous trials, with some patients showing new or progression of extramedullary hematopoietic masses although these could be part of the natural course of the disease.^24^ The scenario raised many questions in the thalassemia community with regards to what the regulatory agencies are looking for when evaluating agents for the treatment of anemia in NTDT. First, improvement of anemia (hemoglobin level) by itself should be considered a clinical benefit considering all the aforementioned evidence indicating the better overall and complication-free survival of individuals with a hemoglobin level >10 g/dL. Anemia is a condition of clinical concern or a marker of one (ineffective erythropoiesis and hemolysis). Proving secondary clinical benefit from ameliorating anemia through an improvement in survival or a reduction in complications is a major challenge because these benefits occur over many years or decades. These benefits can only be assessed in extension studies or through real-world evidence. Measuring the impact on short-term effects like fatigue is also challenging, albeit doable, provided the right tools and endpoints are put in place. This would not be the first time where an index or surrogate marker of disease is used for approvals. Oral iron chelators were approved based on indices of iron overload without demonstrating benefits in organ function, which would again take a long time. Improvement in other surrogate markers of ineffective erythropoiesis could support the idea of long-term clinical benefit, although these are not widely used in common practice.^26^ Denying NTDT patients options to improve anemia (other than through transfusions), and thus potential benefit in the long term, on the basis of suboptimal improvement in short-term outcomes seems short sighted and arbitrary.

We hope to have shed light on a critical unmet need in this patient population and urge colleagues to monitor their NTDT patients closely and regularly for low hemoglobin levels which can deteriorate over time.^27^ Equally, we call on the pharmaceutical industry and regulatory agencies to collaborate on offering a treatment option for ineffective erythropoiesis and anemia associated with NTDT, to mitigate risks of morbidity, mortality, and healthcare resource utilization.

## AUTHOR CONTRIBUTIONS

All authors contributed to manuscript drafting or critical review and final approval for submission.

## DISCLOSURES

KMM reports consultancy fees from Novartis, Celgene Corp (Bristol Myers Squibb), Agios Pharmaceuticals, CRISPR Therapeutics, Vifor Pharma, and Pharmacosmos. ATT reports consultancy fees from Novartis, Celgene Corp (Bristol Myers Squibb), Vifor Pharma, Silence Therapeutics and Ionis Pharmaceuticals; and research funding from Novartis, Celgene Corp (Bristol Myers Squibb), La Jolla Pharmaceutical Company, Roche, Protagonist Therapeutics and Agios Pharmaceuticals. MDC reports consultancy fees from Novartis, Celgene Corp (Bristol Myers Squibb), Vifor Pharma and Ionis Pharmaceuticals; and research funding from Novartis, Celgene Corp (Bristol Myers Squibb), La Jolla Pharmaceutical Company, Roche, Protagonist Therapeutics and CRISPR Therapeutics. OH reports grant support from Alexion Pharmaceuticals, Celgene Corp (Bristol Myers Squibb), and Takeda California. KHMK reports consultancy fees from Agios Pharmaceuticals, Alexion, Apellis, bluebird bio, Celgene Corp (Bristol Myers Squibb), Forma, Pfizer, and Novartis; honoraria from Alexion and Novartis; membership on an advisory committee for Agios Pharmaceuticals and Bioverativ/Sanofi/Sangamo; and research funding from Pfizer. SS reports consultancy fees from Agios Pharmaceuticals, bluebird bio, Bristol Myers Squibb, Forma, and Chiesi; and serving on a clinical trial steering committee for CRISPR/Vertex CTX001 for thalassemia. VV reports consultancy fees from Celgene Corp (Bristol Myers Squibb). JBP reports advisory board fees from bluebird bio, Celgene Corp (Bristol Myers Squibb), Silence Therapeutics, and Vifor Pharma.
